# Synthesis, Biological Evaluation, and Molecular Docking of Tellurium‐Containing Benzothiazole Derivatives as Enzyme Inhibitors of DNA Gyrase and Dihydrofolate Reductase

**DOI:** 10.1155/bmri/9439665

**Published:** 2026-04-16

**Authors:** Olha Haleha, Valeriy Pantyo, Nataliya Korol, Elvira Danko, Mykhailo Onysko

**Affiliations:** ^1^ Department of Organic Chemistry, Educational and Research Institute of Chemistry and Ecology, Uzhhorod National University, Uzhhorod, Ukraine; ^2^ Department of Microbiology, Virology, Epidemiology With the Course of Infectious Diseases, Uzhhorod National University, Uzhhorod, Ukraine; ^3^ Department of Restorative Dentistry, Educational and Scientific Institute of Dentistry and Laboratory Medicine, Uzhhorod National University, Uzhhorod, Ukraine

## Abstract

Tellurium‐containing heterocycles represent an emerging class of organochalcogen compounds with notable redox properties and antimicrobial potential. Incorporation of tellurium into sulfur‐ and nitrogen‐containing frameworks can enhance biological activity through increased polarizability and favorable noncovalent interactions. In this study, a series of novel alkenyl and alkynyl thio‐derivatives of 2‐mercaptobenzothiazole were synthesized via electrophilic heterocyclization with tellurium tetrahalides and evaluated for antibacterial and antibiofilm activity. The resulting benzothiazolium tellurate derivatives were characterized by IR and ^1^H/^13^C NMR spectroscopy, R_F_ values, and elemental analysis. Antibacterial testing against *Staphylococcus aureus* and *Escherichia coli* revealed minimum inhibitory concentration (MIC) values ranging from 31.25 to 250 *μ*g/mL and minimum bactericidal concentration (MBC) values of 62.5–500 *μ*g/mL, with alkynyl‐ and alkenyl‐substituted derivatives displaying the highest activity. Several compounds also demonstrated pronounced inhibition of biofilm formation at sub‐MIC concentrations. Molecular docking studies were performed against bacterial DNA gyrase B and dihydrofolate reductase (DHFR). The most active alkynyl derivative exhibited favorable binding within the ATP‐binding pocket of *S. aureus* gyrase B, whereas selected alkenyl derivatives showed stable accommodation in the active site of *E. coli* DHFR. Predicted binding energies ranged from −4.9 to −6.7 kcal/mol, consistent with moderate but meaningful ligand–enzyme interactions. The results indicate that tellurium‐containing benzothiazolium derivatives constitute a promising exploratory scaffold for antimicrobial and antibiofilm agents. Their activity appears to arise from a combination of enzyme binding, electrophilic reactivity, and enhanced electronic interactions introduced by tellurium incorporation, supporting further investigation of these systems in preclinical antimicrobial research.

## 1. Introduction

The rapid rise of antibiotic‐resistant microorganisms continues to pose a serious global health challenge and drives the ongoing search for new antimicrobial and antibiofilm agents. Among the many classes of bioactive compounds, heterocycles remain especially important because their structural diversity allows effective interaction with a wide range of biological targets. Nitrogen‐containing heterocycles, in particular, have repeatedly demonstrated antifungal and antibacterial potential and are therefore regarded as valuable scaffolds for the development of new pharmacologically active molecules [1, 2].

Within this broad family, benzothiazole derivatives have received sustained attention because of their wide spectrum of biological effects and synthetic versatility. Previous studies have shown that this scaffold is associated with antimicrobial, antiviral, antioxidant, and anticancer activities [3–7]. Of special importance are 2‐mercaptobenzothiazole derivatives, which serve as convenient synthetic intermediates for the introduction of various pharmacophoric substituents capable of modulating biological activity [4, 6].

Recent work has shown that the incorporation of chalcogen‐containing fragments into heterocyclic systems can substantially enhance antimicrobial properties. This tendency is illustrated by the preparation of [1, 3]thiazolo [3,2‐b][1, 2, 4]triazolium salts that displayed antibacterial activity supported by molecular docking data [8], as well as by halogen‐ and chalcogen‐functionalized thiazoloquinazoline derivatives with pronounced bactericidal effects against *Escherichia coli* [9]. Related studies have also demonstrated that benzothiazolium salts are versatile intermediates capable of generating structurally diverse heterocyclic systems [10]. Thus, the combination of benzothiazole cores with chalcogen‐containing fragments represents a promising approach to the design of new antimicrobial agents.

Particular interest has recently focused on organotellurium compounds because of their distinctive electronic properties and their ability to participate in redox processes and coordinate with thiol‐containing biomolecules. Chalcogen‐substituted thiazole and benzothiazole derivatives have been recognized as medicinally relevant structures [11], whereas electrophilic cyclization has emerged as an efficient route to condensed heterocycles containing chalcogen atoms [12]. The synthetic value of electrophilic heterocyclization has been especially emphasized for alkynes bearing heteroatom‐containing substituents since this strategy enables rapid access to diverse heterocyclic frameworks [13].

The scope of electrophilic heterocyclization has continued to expand in the synthesis of biologically active compounds. Efficient routes to mononuclear heterocycles have been developed on this basis [14], and modern N‐halo reagents have further broadened the synthetic possibilities for heterocycle construction [15]. In biological studies, halogen‐containing azaheterocycles were shown to affect the sensitivity of *Staphylococcus aureus* to antimicrobial agents [16]. In parallel, unexpected tellurohalogenation of N‐alkynyl heterocycles has highlighted the strong electrophilic character of tellurium tetrahalides in cyclization processes [17].

Further insight into the chemistry of tellurium‐containing compounds has been provided by structural and mechanistic studies. Halogen‐dependent trends in the structure and reactivity of tellurium‐based zwitterions have been demonstrated using combined spectroscopic and theoretical approaches [18]. The formation of hexahalotellurate salts under protonating conditions has also been described [19]. Electrophilic intramolecular cyclization of unsaturated thioethers has afforded dihydrothiazoloquinazoline derivatives, showing that substituent effects strongly influence reaction pathways [20]. Related transformations have confirmed that tellurium tetrahalides can efficiently promote the cyclization of alkynyl thioethers to generate functional heterocycles [21].

The biological potential of organotellurium compounds has also become increasingly evident. Pyrazole‐based tellurium derivatives have shown antibacterial activity against pathogenic microorganisms [22]. Organic–inorganic hexabromotellurate hybrid materials have been characterized in detail, revealing structural and electronic features relevant to bioactivity [23]. Functional thiazolo‐triazole derivatives have further confirmed the antimicrobial promise of chalcogen‐containing heterocycles [24], whereas hybrid hexachlorotellurate systems have demonstrated antibacterial properties supported by theoretical analysis [25]. In addition, cationic thiazolotriazolium surfactants have shown the ability to stabilize noble metal nanoparticles, illustrating the broader functional potential of such systems [26].

Interest in tellurium chemistry is also supported by its wider pharmacological relevance. Synthetic organoselenium and organotellurium compounds are known to interact with thiol‐containing proteins and to influence oxidative stress pathways [27]. Electrophilic chalcogenation strategies have enabled access to biologically active isoxazoline derivatives [28], and oxime‐based methods continue to expand the synthetic toolbox for heterocycle construction [29]. More recently, electrocatalytic multicomponent reactions have provided tellurium‐containing oxazolidinones with promising anticancer properties [30].

From a medicinal standpoint, tellurium compounds have also attracted attention as potential therapeutic agents. The tellurium‐based prodrug AS101 has been widely studied as an immunomodulator capable of interacting with thiol‐containing enzymes [31]. Replacement of halide ligands in related analogues was shown to markedly influence their stability and reactivity [32], whereas structural studies of hexabromotellurate salts further demonstrated that the ligand environment plays a decisive role in determining the physicochemical behavior of tellurium‐containing species [33].

At the same time, the antimicrobial potential of benzothiazole derivatives remains well established. Benzothiazoles have been recognized for their ability to inhibit microbial enzymes and disrupt bacterial viability [34]. Experimental studies have confirmed both antibacterial and antibiofilm activities for substituted benzothiazoles [35], and earlier investigations also showed that heterocyclic compounds and phytochemicals can inhibit antibiotic‐resistant bacteria [36].

Modern antimicrobial research increasingly relies on the combination of experimental and computational methods. Molecular modeling and docking studies have provided valuable insight into the interactions between benzothiazole derivatives and bacterial enzymes [37]. Thiazole‐based chalcones have likewise been identified as inhibitors of dihydrofolate reductase (DHFR), an important antibacterial target [38]. Structural information for such studies is commonly obtained from the Protein Data Bank (PDB) [39], while validation tools for metal‐binding sites help ensure the reliability of docking analyses involving metalloid‐containing systems [40].

Despite this progress, benzothiazolium salts bearing long‐chain unsaturated thio substituents and tellurium‐based counterions remain insufficiently explored. In particular, the influence of the type of unsaturation, namely alkene versus alkyne, together with the identity of the tellurium halide, on the formation, structure, and biological activity of these compounds has not been systematically investigated. For this reason, the present work focuses on the synthesis of a new series of tellurium‐containing benzothiazolium salts via electrophilic heterocyclization using tellurium tetrahalides, TeCl_4_ and TeBr_4_, followed by evaluation of their antibacterial and antibiofilm properties.

The synthesized compounds were tested against Gram‐positive *S. aureus* and Gram‐negative *E. coli*. To clarify the molecular basis of their activity, molecular docking studies were carried out against key bacterial enzymes, including DHFR and other essential targets.

## 2. Materials and Methods

### 2.1. Chemistry

All reagents and solvents were of analytical grade and used without further purification. Melting points were determined using a Stuart SMP30 apparatus. ^1^H NMR (400 MHz) and ^13^C NMR (100 MHz) spectra were recorded in DMSO‐d_6_ using tetramethylsilane (TMS) as an internal standard on a Varian VXR‐400 spectrometer. Elemental analyses were performed on an Elementar Vario MICRO cube analyzer, and the obtained values were in good agreement with the calculated data. NMR spectra (^1^H, ^13^C) for all synthesized compounds are provided in the Supporting Information.

#### 2.1.1. General Procedure for the Synthesis of Hexahalotellurates 3‐6

A solution of hexahalotelluric acid was prepared by dissolving TeO_2_ (1.4 mmol) in a sixfold molar excess of the corresponding hydrohalic acid (HBr, *ρ* = 1.30 g/cm^3^, or HCl, *ρ* = 1.19 g/cm^3^). To this freshly prepared acid solution, 1.4 mmol of the appropriate thioether (1 or 2), dissolved in 10–15 mL of chloroform or acetic acid, was added dropwise under continuous stirring at room temperature. The reaction mixture was stirred for 24 h, after which the solid product was filtered, washed with chloroform or acetic acid, and air‐dried.

##### 2.1.1.1. Hexabromotellurate of 2‐Butynylthiobenzothiazolium 3.

Yield: 1.01 g (70%). Orange solid. m.p. 255°C–256°C. Rf 0.73 (EtOAc : AcOH = 3 : 1). ^1^H NMR (400 MHz, DMSO‐d_6_) *δ* (ppm): 2.73 (td, 2H, *J* = 4 Hz), 2.96 (t, 1H, *J* = 4 Hz), 3.52 (t, 2H, *J* = 8 Hz), 7.36 (t, 1H, *J* = 8 Hz), 7.46 (t, 1H, *J* = 8 Hz), 7.87 (d, 1H, *J* = 8 Hz), 8.01 (d, 1H, *J* = 8 Hz). Elemental analysis for C_22_H_20_Br_6_N_2_S_4_Te: calcd C 23.59, H 1.95, N 2.73, Br 46.78, S 12.48; found C 22.85, H 1.72, N 2.59, Br 48.01, S 11.96.

##### 2.1.1.2. Hexachlorotellurate of 2‐Butynylthiobenzothiazolium 4.

Yield: 0.77 g (72%). Yellow solid. m.p. 215°C–220°C. Rf 0.67 (EtOAc : AcOH = 3 : 1). ^1^H NMR (400 MHz, DMSO‐d_6_) *δ* (ppm): 2.72 (td, 2H, *J* = 4 Hz), 2.96 (t, 1H, *J* = 4 Hz), 3.52 (t, 2H, *J* = 8 Hz), 7.36 (t, 1H, *J* = 8 Hz), 7.46 (t, 1H, *J* = 8 Hz), 7.87 (d, 1H, *J* = 8 Hz), 8.01 (d, 1H, *J* = 8 Hz). Elemental analysis for C_22_H_20_Cl_6_N_2_S_4_Te: calcd C 31.88, H 2.64, N 3.70, Cl 28.06, S 16.86; found C 30.65, H 2.49, N 3.39, Cl 29.35, S 16.05.

##### 2.1.1.3. Hexabromotellurate of 2‐Pentenylthiobenzothiazolium 5.

Yield: 0.99 g (68%). Orange solid. m.p. 96°C–97°C. Rf 0.85 (EtOAc : AcOH = 3 : 1). ^1^H NMR (400 MHz, DMSO‐d_6_) *δ* (ppm): 1.86 (m, 2H), 2.19 (m, 2H), 3.35 (t, 2H, *J* = 8 Hz), 5.00 (d, 1H, *J* = 12 Hz), 5.07 (d, 1H, *J* = 16 Hz), 5.83 (m, 1H), 7.35 (t, 1H, *J* = 8 Hz), 7.45 (t, 1H, *J* = 8 Hz), 7.85 (d, 1H, *J* = 8 Hz), 8.00 (d, 1H, *J* = 8 Hz). Elemental analysis for C_23_H_24_Br_6_N_2_S_4_Te: calcd C 23.22, H 2.30, N 2.69, Br 46.07, S 12.28; found C 22.86, H 2.19, N 2.44, Br 47.28, S 11.86.

##### 2.1.1.4. Hexachlorotellurate of 2‐Pentenylthiobenzothiazolium 6.

Yield:0.77 g (71%). Yellow solid. m.p. 115°C–117°C. Rf 0.76 (EtOAc : AcOH = 3 : 1). ^1^H NMR (400 MHz, DMSO‐d_6_) *δ* (ppm): 1.87 (m, 2H), 2.19 (m, 2H), 3.35 (t, 2H, *J* = 8 Hz), 5.01 (d, 1H, *J* = 12 Hz), 5.07 (d, 1H, *J* = 16 Hz), 5.83 (m, 1H), 7.35 (t, 1H, *J* = 8 Hz), 7.45 (t, 1H, *J* = 8 Hz), 7.84 (d, 1H, *J* = 8 Hz), 8.00 (d, 1H, *J* = 8 Hz). Elemental analysis for C_23_H_24_Cl_6_N_2_S_4_Te: calcd C 31.23, H 3.10, N 3.61, Cl 27.48, S 16.52; found C 30.84, H 2.95, N 3.39, Cl 28.14, S 15.96.

#### 2.1.2. General Procedure for the Synthesis of Benzo[4, 5]thiazolo[2,3‐b][1, 3]thiazinium Halides 7, 8

To a solution of thioether 1 (1.4 mmol) in 10–15 mL of glacial acetic acid, a freshly prepared solution of hexahalotelluric acid (from 1.4 mmol TeO_2_ and sixfold molar excess of HBr or HCl) was added. The reaction mixture was stirred at 55°C–60°C for 24 h. The resulting precipitate was filtered, washed with acetic acid, and air‐dried.

##### 2.1.2.1. 4‐[(Tetrabromo‐*λ* ^5^‐tellanyl)methylidene]‐3,4‐dihydro‐2*H*‐benzo[4, 5]thiazolo[2,3‐*b*][1, 3]thiazinium‐5 7.

Yield: 0.57 g (61%). Orange solid. m.p. 210°C–211°C. Rf 0.63 (EtOAc : AcOH = 3 : 1). ^1^H NMR (400 MHz, DMSO‐d_6_) *δ* (ppm): 3.64 (m, 4H), 7.37 (t, 1H, *J* = 8 Hz), 7.47 (t, 1H, *J* = 8 Hz), 7.72 (s, 1H), 7.87 (d, 1H, *J* = 8 Hz), 8.02 (d, 1H, *J* = 8 Hz). ^13^C NMR (100 MHz, DMSO‐d_6_) *δ* (ppm): 32.0 (СH_2_), 39.0 (СH_2_), 121.7 (C_benz_), 122.3 (C_benz_), 124.9 (C_benz_), 126.8(C_benz_), 131.3 (C_thiazol_‐S), 132.8 (=CH‐Te), 135.1 (C_thiazol_‐N^+^), 153.1 (C=N^+^), 166.4 (C_thiazin_‐N^+^). Elemental analysis for C_11_H_9_Br_4_NS_2_Te: calcd C 19.82, H 1.36, N 2.10, Br 47.95, S 9.62; found C 17.65, H 1.33, N 1.86, Br 49.55, S 8.91.

##### 2.1.2.2. 4‐[(Tetrachloro‐*λ* ^5^‐tellanyl)methylidene]‐3,4‐dihydro‐2*H*‐benzo[4, 5]thiazolo [2,3‐*b*][1, 3]thiazinium‐5 8.

Yield: 0.44 g (64%). Yellow solid. m.p. 175‐177°C. Rf 0.41 (EtOAc : AcOH = 3 : 1). ^1^H NMR (400 MHz, DMSO‐d_6_) *δ* (ppm): 3.53 (d, 2H, *J* = 8 Hz), 3.62 (d, 2H, *J* = 8 Hz), 7.36 (t, 2H, *J* = 8 Hz), 7.46 (t, 1H, *J* = 8 Hz), 7.87 (d, 1H, *J* = 8 Hz), 8.01 (d, 1H, *J* = 8 Hz). ^13^C NMR (100 MHz, DMSO‐d_6_) *δ* (ppm): 31.1 (СH_2_), 36.1 (СH_2_), 121.7 (C_benz_), 122.3 (C_benz_), 124.9 (C_benz_), 126.8(C_benz_), 135.0 (C_thiazol_‐S), 140.5 (=CH‐Te), 142.9 (C_thiazol_‐N^+^), 153.1 (C=N^+^), 166.4(C_thiazin_‐N^+^). Elemental analysis for C_11_H_9_Cl_4_NS_2_Te: calcd C 27.03, H 1.86, N 2.87, Cl 29.02, S 13.12; found C 22.67, H 2.11, N 2.82, Cl 30.48, S 12.11.

### 2.2. Biological Activity

#### 2.2.1. Antimicrobial Activity

The antimicrobial activity of the synthesized compounds was evaluated in the Microbiology, Virology, Epidemiology With the Course of Infectious Diseases Department, State Higher Educational Institution “Uzhhorod National University.” Antibacterial and antifungal properties were determined by the serial dilution method to establish the minimum inhibitory concentration (MIC) and minimum bactericidal concentration (MBC) values, in accordance with international recommendations of CLSI M07 and EUCAST guidelines for in vitro antimicrobial testing [41, 42].

Microbial test cultures included *S. aureus*, *Candida albicans*, *Klebsiella pneumoniae*, *E. coli*, and *Pseudomonas aeruginosa.* Microbial suspensions were prepared from 24‐h cultures on appropriate media, adjusted to 0.5 McFarland standard (≈1.5 × 10^8^ CFU/mL). Each well of a sterile 96‐well plate received 10 *μ*L of the standardized microbial suspension and 190 *μ*L of compound solution prepared in sterile broth.

Control groups were (1) microbial growth control—inoculated medium without compound (to verify viability); (2) compound sterility control—compound solution without inoculum (to exclude contamination); and (3) solvent control—medium with solvent only at the working concentration (to rule out solvent antimicrobial effect).

Plates were incubated at 37°C for 24 h, followed by titration via serial twofold dilutions and reseeding onto selective agar media (Nutrient agar, Sabouraud, Endo, and Mannitol‐salt agar). After 24–48 h of incubation, the growth or absence of colonies was assessed visually to determine MIC and MBC. All procedures were conducted under sterile conditions; sterility of the preparations was confirmed bacteriologically by plating 10‐*μ*L aliquots on meat‐peptone agar.

#### 2.2.2. Antibiofilm Activity

Compounds **4** and **8** were further evaluated for their antibiofilm activity using a modified microtiter‐plate assay as described by Azeredo et al. and Djordjevic et al. [43, 44].

Four‐day‐old biofilms of *S. aureus*, *C. albicans*, *E. coli*, and *K. pneumoniae* were cultivated in 96‐well plates by inoculating 190 *μ*L of sterile broth with 10 *μ*L of a 0.5 McFarland microbial suspension. Plates were sealed and incubated at 37°C for 96 h to allow mature biofilm formation.

After incubation, planktonic cells were removed, and wells were rinsed twice with sterile distilled water. Then, 200 *μ*L of compound solution (final concentration = 1000 *μ*g/mL) was added to each well, followed by 24 h incubation at 37°C. Subsequently, wells were washed three times with 200 *μ*L of sterile distilled water, filled with 200‐*μ*L sterile water, and the biofilms were carefully scraped from the well surfaces using pipette tips.

A total of 20‐*μ*L aliquots of the obtained suspensions were serially diluted (10^−1^–10^−4^) in Eppendorf tubes containing 180‐*μ*L sterile water or saline. Subsequently, 10‐*μ*Lsamples from each dilution were plated on appropriate nutrient agar and incubated 24 h at 37°C. The absence of microbial growth was interpreted as complete biofilm inhibition. Five‐day‐old untreated biofilms served as negative controls.

These procedures enabled quantitative comparison of antibiofilm efficacy across the tested strains and ensured reproducibility under standardized conditions.

### 2.3. Docking Procedure

Docking simulations were performed using AutoDock Vina software [45], an open‐source tool recognized for its efficiency and accuracy in predicting ligand–receptor interactions. Protein structures (5BTC [46], 5MMN [47], 2W9S [48], and 1RX2 [49]) were retrieved from the PDB [37] and preprocessed in BIOVIA Discovery Studio Visualizer 2021 [50] by removing cocrystallized ligands and water molecules, adding hydrogen atoms and Gasteiger charges, and defining the grid box around the cofactor or active site.

Ligand structures of the tested compounds were constructed and energy‐minimized in Avogadro [51] using the MMFF94 force field to obtain the lowest‐energy conformers. Each docking run was executed using a Genetic‐Algorithm‐based search method, generating 10 distinct poses per ligand–protein complex. Binding poses were ranked according to predicted affinity (kcal/mol).

Postdocking analyses—including visualization of hydrogen bonding, *π*–*π* stacking, hydrophobic interactions, and mapping of amino‐acid contacts—were carried out using BIOVIA Discovery Studio [50]. Binding energies and interaction profiles were compared across all target proteins to identify the most probable inhibitory conformations.

## 3. Results and Discussion

### 3.1. Synthesis

**Scheme 1:**
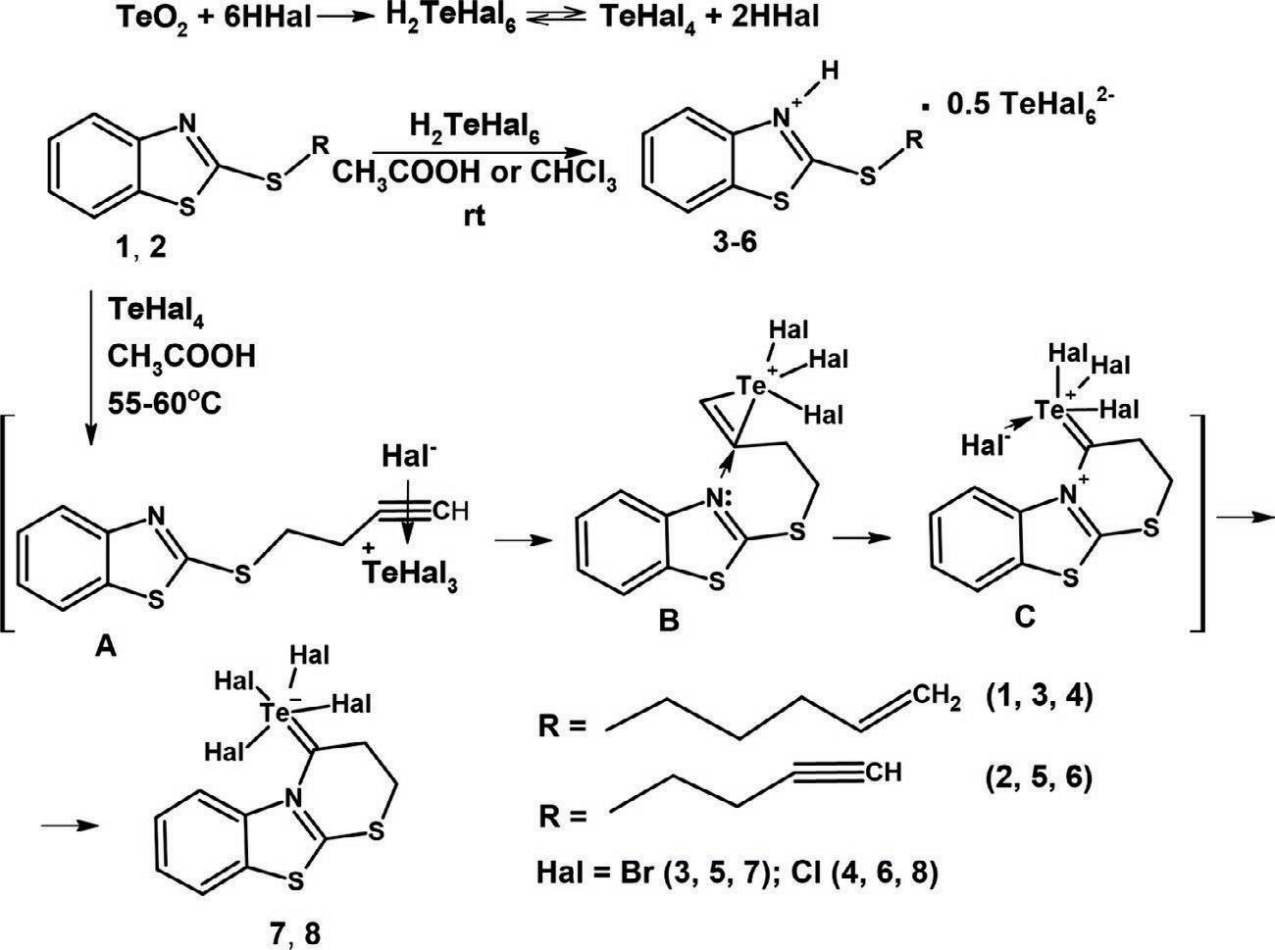
Synthesis of benzothiazolium hexahalotellurate salts 3â€“6 and Te(IV)‐induced cyclization products 7 and 8.

The synthetic approach toward the target tellurium‐containing heterocycles was based on the electrophilic heterocyclization of long‐chain alkyl‐ and alkenylthio derivatives of 2‐mercaptobenzothiazole (Compounds **1 and 2**) with tellurium tetrahalides generated in situ (Scheme 1). The electrophilic system TeO_2_ + 6 HHal (Hal = Br, Cl) was employed as a classical reagent mixture capable of providing both proton‐ and tellurium‐induced cyclization conditions. Depending on solvent polarity and reaction temperature, the transformation proceeded through distinct pathways leading either to ionic benzothiazolium salts or to condensed heterocyclic products.

When the reactions were carried out in chloroform or acetic acid at room temperature, the interaction of thioethers (**1 and 2**) with the electrophilic tellurium system afforded benzothiazolium hexahalotellurate complexes (**3–6**), isolated as stable crystalline compounds containing half an equivalent of the hexahalotellurate anion (0.5 TeHal_6_^2−^). Their formation indicates predominance of proton‐induced electrophilic cyclization under low‐temperature and moderately polar conditions. In contrast, performing the same reactions in acetic acid at 55°C–60°C resulted in condensed tellurium‐induced heterocyclization products (**7 and 8**), suggesting that elevated temperature and increased medium polarity favor direct electrophilic activation by TeX_4_ followed by intramolecular ring closure.

Mechanistically, the electrophilic system generated from TeO_2_ and hydrogen halides can be described as an equilibrium between protonated hexahalotellurate species and reactive tellurium(IV) tetrahalides, the position of which depends strongly on solvent polarity and temperature. Tellurium oxide in the presence of excess hydrohalic acid forms hexahalotelluric acid, which exists in equilibrium with tellurium tetrahalides; increasing solvent polarity and temperature shifts this equilibrium toward formation of TeX_4_. Under mild conditions, protonated hexahalotellurate species prevail and promote protonation of alkyl‐unsaturated benzothiazole thioethers in chloroform or acetic acid, leading to Salts **3–6**. At elevated temperatures, molecular TeX_4_ acts as a dominated electrophile and reacts directly with the unsaturated substituent, giving rise to condensed Products **7** and **8**.

The electrophilic heterocyclization is proposed to begin with the addition of a Te(IV) halide to the C = C or C ≡ C bond of the Substrate **A**, generating a cyclic telluronium *π*‐Complex **B** (or three‐center telluronium cation), analogous to a halonium‐type intermediate. Similar telluronium intermediates and unexpected tellurohalogenation pathways have been reported for N‐alkynyl and N‐alkenyl heterocycles, supporting the plausibility of this activation step [17]. Subsequent intramolecular nucleophilic attack by the thiazole nitrogen or sulfur atom, as shown at the intermediate **C**, leads to ring closure and formation of condensed benzothiazolium frameworks. Final stabilization occurs through ion pairing with the [TeX_6_]^2−^ counter‐anion or via internal Lewis acid–base interaction, resulting in ionic or zwitterionic products.

Solvent effects play a decisive role in directing the reaction pathway. In polar or protic media (e.g., acetic acid/HCl), protonation and stabilization of the hexahalotellurate anion [TeX_6_]^2−^ dominate, favoring benzothiazolium tellurate salts. This behavior agrees with established stability and speciation patterns of hypervalent tellurium compounds in aqueous and protic environments [52]. The nature of the unsaturated substituent further influences the outcome: alkynes, owing to higher *π*‐reactivity, facilitate cyclization pathways (e.g., 5‐exo‐dig closure), whereas alkenes more readily stabilize benzothiazolium salt structures. Halogen identity also modulates reactivity, as TeBr_4_ typically displays higher electrophilic activity due to weaker Te‐Br bonds and better leaving ability of Br^-^, whereas TeCl_4_ is more prone to hydrolysis to [TeOCl_3_]^−^ and tends to form more stable tellurate salts. These observations are consistent with recent regiospecific and stereospecific studies on TeBr_4_‐mediated electrophilic additions to terminal alkynes [53] and broader analyses of condition‐dependent selectivity in Te(IV) reactions with unsaturated substrates [30–32].

The structures of all synthesized products were confirmed using a comprehensive set of spectroscopic techniques, including ^1^H NMR, ^13^C NMR, FT‐IR spectroscopy, elemental analysis, and high‐resolution ESI‐TOF mass spectrometry. Representative FT‐IR bands (KBr, cm^−1^) include strong C = N and C = C stretches at 1600–1700 cm^−1^ and Te‐O bands at 600–800 cm^−1^, whereas Te‐Cl/Te‐Br vibrations occur below 300 cm^−1^ and therefore are not observed in the mid‐IR region. High‐resolution ESI‐TOF spectra confirmed molecular masses within 5 ppm. The combined spectroscopic and analytical data provide consistent evidence for the proposed structures and support the presence of electrophilic tellurium centers and *π*‐conjugated unsaturated fragments that underpin the observed reactivity patterns.

### 3.2. Biological Activity

The biological evaluation of the synthesized tellurium‐containing benzothiazole derivatives (**3–8**) aimed to assess their potential as antimicrobial and antibiofilm agents. Given the established bioactivity of benzothiazole and chalcogen‐substituted heterocycles, the compounds were screened against clinically relevant microorganisms, including Gram‐positive (*S. aureus*) and Gram‐negative bacteria (*K. pneumoniae*, *E. coli*, and *P. aeruginosa*), as well as the opportunistic fungus *C. albicans*.

Antimicrobial activity was determined using the serial dilution method to measure both MIC and MBC, within a concentration range of 250–15.625 *μ*g/mL, following international standards [41, 42]. All experiments were performed using five independent clinical isolates for each species, and results are presented as mean values. To enable benchmarking, reference antimicrobial agents (fluconazole, levofloxacin, and lincomycin) were tested under identical experimental conditions. The results are summarized in Table 1.

**Table 1:** Antimicrobial activity (MIC and MBC, *μ*g/mL) of tellurium‐containing benzothiazole Derivatives 3–6 and referent drugs.

Agent/compound	*C. albicans* MIC/MBC	*S. aureus* MIC/MBC	*K. pneumoniae* MIC/MBC	*E. coli* MIC/MBC	*P. aeruginosa* MIC/MBC
**Compound 3**	31.25/250	250/250	250/500	125/125	250/500
**Compound 4**	31.25/250	62.5/125	250/500	31.25/62.5	250/500
**Compound 5**	31.25/500	250/250	125/250	62.5/125	125/500
**Compound 6**	62.5/250	62.5/125	250/500	62.5/125	250/500
**Compound 7**	31.25/250	250/250	125/500	125/125	250/500
**Compound 8**	31.25/125	125/250	250/500	62.5/125	125/500
**Fluconazole**	< 15.625/ND	ND	ND	ND	ND
**Levofloxacin**	ND	< 15.625/< 15.625	< 15.625/< 15.625	< 15.625/< 15.625	< 15.625/< 15.625
**Lincomycin**	ND	< 15.625/< 15.625	250/> 250	>250/>250	> 250/> 250

Compounds **4** and **6** emerged as the most potent inhibitors, particularly against *S. aureus* and *C. albicans*, exhibiting MIC values as low as 31.25 *μ*g/mL. Compounds **3, 5**, and **7** displayed moderate yet consistent antimicrobial effects, whereas Compound **8** showed slightly lower activity, though still within the effective range typical of exploratory organic heterocyclic antimicrobials. As expected, the synthesized compounds were less potent than reference antibiotics such as levofloxacin, which exhibited MIC values below 15.625 *μ*g/mL; however, the observed activity is notable for a new organotellurium scaffold.

As expected, Gram‐negative strains (*E. coli* and *K. pneumoniae*) were generally more resistant, consistent with the protective role of the outer membrane that restricts penetration of lipophilic molecules [34, 49]. Gram‐positive bacteria and fungi, which lack this barrier, were more susceptible due to the accessibility of their peptidoglycan‐ or ergosterol‐rich cell walls. These features promote electrostatic and hydrophobic interactions with the benzothiazolium–tellurium moiety. The incorporation of tellurium appears to enhance antimicrobial efficacy through synergistic effects of electrophilicity, redox activity, and increased molecular polarization.

The biological behavior of Compounds **3–8** is governed by a combination of electronic and steric factors, primarily the type of unsaturated substituent, the halogen atom, and the ionic or condensed nature of the molecule. The electrophilic tellurium center, defined by the Te‐Hal bond (TeCl_4_^−^ or TeBr_4_^−^), imparts strong electrophilicity and redox reactivity, enabling interactions with thiol‐ and selenol‐containing enzyme residues such as those in thioredoxin reductase, glutathione reductase, and DHFR. These interactions likely disturb redox homeostasis and trigger oxidative stress, a mechanism similar to that reported for other chalcogen‐containing heterocycles [5, 8, 54].

Both alkenyl (**3** and **4**) and alkynyl (**5** and **6**) complexes demonstrated greater antimicrobial activity than their saturated counterparts. The C ≡ C‐containing compounds (**5** and **6**) proved slightly more potent than the C = C analogues (**3** and **4**), reflecting enhanced *π*‐electron delocalization, increased rigidity, and improved ability to form *π*–*π* stacking or charge–transfer interactions with aromatic residues in enzymes or nucleic acids. Molecular docking supports this trend, indicating that the alkynyl Derivative **6** fits optimally into the ATP‐binding groove of DNA gyrase B (GyrB), whereas the alkenyl Analogue **4** is well accommodated in the NADPH pocket of DHFR.

The halogen effect also played a decisive role: chlorinated derivatives (**4**, **6**, and **8**) were generally more active than their brominated counterparts (**3**, **5**, and **7**). This difference may arise from chlorine′s smaller ionic radius, higher electronegativity, and stronger Te‐Cl bond, which enhance molecular polarization and potentially improve membrane penetration. Moreover, condensed heterocycles (**7** and **8**) exhibited moderate yet broad‐spectrum activity, likely due to increased lipophilicity, whereas ionic complexes (**3–6**) showed greater affinity toward negatively charged microbial surfaces, translating into stronger activity against Gram‐positive bacteria and fungi.

Compounds **4** and **8** were further evaluated for antibiofilm activity against mature, 4‐day‐old biofilms of *S. aureus*, *C. albicans*, *E. coli*, and *K. pneumoniae* using a microtiter plate–based assay [43, 44]. Biofilm viability was quantified by CFU enumeration after mechanical disruption and serial dilution of biofilm scrapings, rather than by crystal violet staining. At a concentration of 1000 *μ*g/mL, both derivatives resulted in complete eradication of viable biofilm‐embedded cells (no detectable CFU growth) after 24 h of exposure.

This pronounced antibiofilm activity can be attributed to the dual lipophilic–electrophilic nature of the Te‐benzothiazolium framework, which facilitates penetration of the extracellular polymeric substance (EPS) and promotes oxidative destabilization of biofilm components. The presence of unsaturated substituents (C = C, C ≡ C) further enhances *π*‐conjugation and surface adsorption, facilitating disruption of protein‐polysaccharide crosslinks and detachment of sessile cells. Due to total eradication observed in all experimental series, statistical comparison between treated and control groups was not applicable.

In summary, tellurium‐containing benzothiazole derivatives—particularly the alkenyl (**3** and **4**) and alkynyl (**5** and **6**) analogues—exhibited notable antibacterial, antifungal, and antibiofilm activities in vitro. Their efficacy is closely linked to unsaturation degree, chlorine coordination, and the presence of electrophilic Te‐Hal centers. The combined effects of redox reactivity, electrophilicity, and lipophilicity provide a rational basis for further molecular optimization. Together with molecular docking insights, these findings identify these compounds as promising exploratory scaffolds for the development of multifunctional organotellurium‐based antimicrobial agents.

### 3.3. Molecular Docking of Tellurium‐Containing Benzothiazole Derivatives

Molecular docking simulations were performed to rationalize the antibacterial and antibiofilm behavior of the newly synthesized tellurium‐containing benzothiazolium Derivatives **4** (thiopentenyl) and **6** (thiobutynyl). The computational study is aimed at (i) identifying plausible enzymatic targets responsible for the observed biological effects, (ii) characterizing key noncovalent interactions governing affinity and selectivity, and (iii) correlating the in silico results with the experimental structure–activity relationships established through microbiological assays.

Two validated antibacterial enzymes were selected as molecular targets: DNA GyrB and DHFR. DNA gyrase catalyzes ATP‐driven negative supercoiling of DNA, an essential step in replication and transcription, whereas DHFR mediates the NADPH‐dependent reduction of dihydrofolate to tetrahydrofolate—critical for purine and thymidylate biosynthesis. Inhibition of either enzyme leads to bacteriostasis or cell death, and simultaneous blockade of both pathways is a recognized strategy for minimizing resistance development [55–58].

To represent both Gram‐positive and Gram‐negative systems, four crystallographic models were employed: *S. aureus* GyrB (2XCR), *E. coli* GyrB (3G7E), *S. aureus* DHFR (3SQY), and *E. coli* DHFR (3SFM).

Table 2 summarizes the calculated binding affinities and key interactions between the compounds and their respective targets.

**Table 2:** Molecular docking interactions and binding affinities of the compounds with target proteins.

Protein ID	Protein (target)	Ligand	Affinity (kcal/mol)	Hydrogen bonds (residue → ligand, Å)	Hydrophobic contacts (alkyl/*π*‐alkyl, Å)	*π* interactions (*π*–*π*/cation‐*π*, Å)
3G7E	*E. coli* DNA gyrase B	Compound **4**	−6.7	—	VAL120 5.49; VAL167 5.09; ILE78 4.72 and 5.49; VAL43 4.07; MET95 5.47	Cation‐*π* with aromatic residues
2XCR	*S. aureus* DNA gyrase B	Compound **6**	−6.5	ARG601 2.07; LEU603 3.65	ARG601 5.15; LEU603 5.16	*π*‐alkyl, *π*‐cation stabilization
2XCR	*S. aureus* DNA gyrase B	Compound **4**	−6.5	PRO598 2.56	LEU603 4.85	TYR538 *π*‐alkyl 4.61
3SQY	*S. aureus* DHFR	Compound **4**	−6.3	PHE93 1.95	ILE15 3.99; VAL32 4.9–5.5	PHE99 *π*‐*π* 4.6‐4.9
3SQY	*S. aureus* DHFR	Compound **6**	−6.2	THR47 1.86	ILE15 4.39; LYS46 4.78	PHE99 *π*‐alkyl 4.96
3SFM	*E. coli* DHFR	Compound **4**	−4.9	—	PRO52 4.39; ALA22 5.00	TRP45 *π*–*π* 3.78‐4.63

Both tellurium derivatives successfully occupied the ATP‐binding pocket of GyrB, overlapping with the aminocoumarin/fluoroquinolone binding site. Compound **4** (thiopentenyl) displayed a binding affinity of −6.5 kcal/mol in *S. aureus* GyrB. Its linear thio‐alkenyl chain fitted neatly into the narrow hydrophobic cleft formed by VAL43, ILE78, VAL120, and MET95, exhibiting excellent van der Waals complementarity. The benzothiazolium cation engaged ARG601 through electrostatic and cation–*π* interactions, whereas the thioether bridge contributed C–H···O hydrogen bonding with LEU603 (3.64 Å) and a polar contact with ARG601 (2.08 Å), stabilizing the complex along the catalytic hinge.

Compound **6** (thiobutynyl) demonstrated the strongest binding, with a score of −6.7 kcal/mol in *E. coli* GyrB. The rigid, sp‐hybridized C ≡ C fragment inserted deeply into the hydrophobic channel bounded by VAL120, VAL167, ILE78, and PRO79 (4.1–5.3 Å). *π*‐stacking with phenylalanine residues and dispersion interactions with sulfur‐rich MET95 were reinforced by the high polarizability of tellurium.

**Figure 1:**
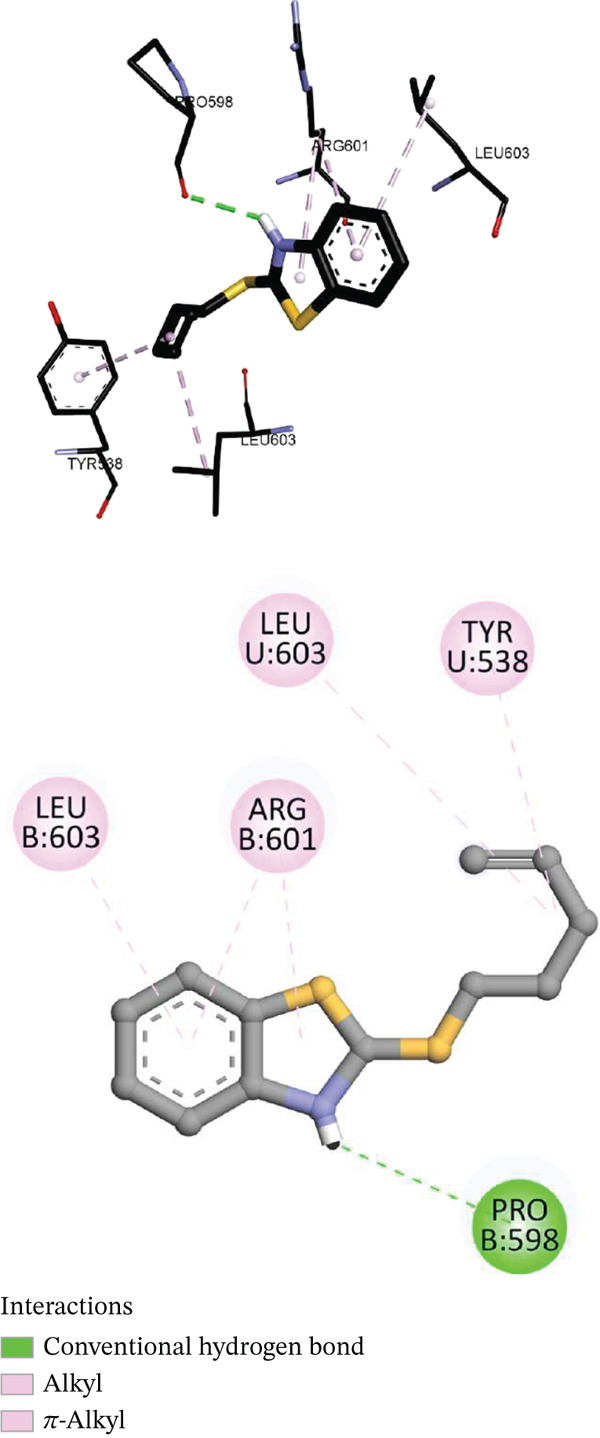
3D and 2D representations of Compound **4** interacting with *S. aureus* DNA gyrase B (PDB ID 2XCR).

Figure 1 can illustrate the 3D and 2D interactions of Compound **4** with *S. aureus* GyrB (PDB 2XCR), highlighting the hydrogen bonds with ARG601 and LEU603 and extensive hydrophobic contacts within the ATP pocket.

This inhibition mode corresponds to ATP‐site occupancy, preventing hydrolysis and subsequent DNA supercoiling. Docking orientations were reminiscent of GSK299423 [55, 56], but the inclusion of tellurium provided distinctive electrostatic and charge‐transfer interactions. The heavy chalcogen′s extended d‐orbitals facilitate *σ*‐hole and polarization effects with carbonyl and sulfur donors, which may account for the observed broad‐spectrum antibacterial activity [59, 60].

Both ligands localized near the NADPH cofactor pocket of DHFR. In *S. aureus* DHFR (3SQY), Compound **6** (thiobutynyl) achieved a binding energy of −6.3 kcal/mol, forming a hydrogen bond with PHE93 (1.95 Å) and *π*–*π* stacking with PHE99 (4.7 Å). Hydrophobic contacts with ILE15, LEU21, VAL32, and ALA8 further stabilized the complex. Compound **4** (thiopentenyl) displayed a similar affinity (−6.2 kcal/mol), anchored by a hydrogen bond with THR47 (1.88 Å) and *π*‐alkyl interactions involving LYS46 and ILE15.

Within *E. coli* DHFR (3SFM), both compounds occupied the smaller hydrophobic cavity adjacent to TRP45. Compound **6** established multiple *π*–*π* stacking interactions (3.8‐4.6 Å) and *π*‐alkyl contacts with PRO52 and ALA22, yielding an overall binding energy of −4.9 kcal/mol, consistent with the shallower, solvent‐exposed nature of the bacterial pocket.

**Figure 2:**
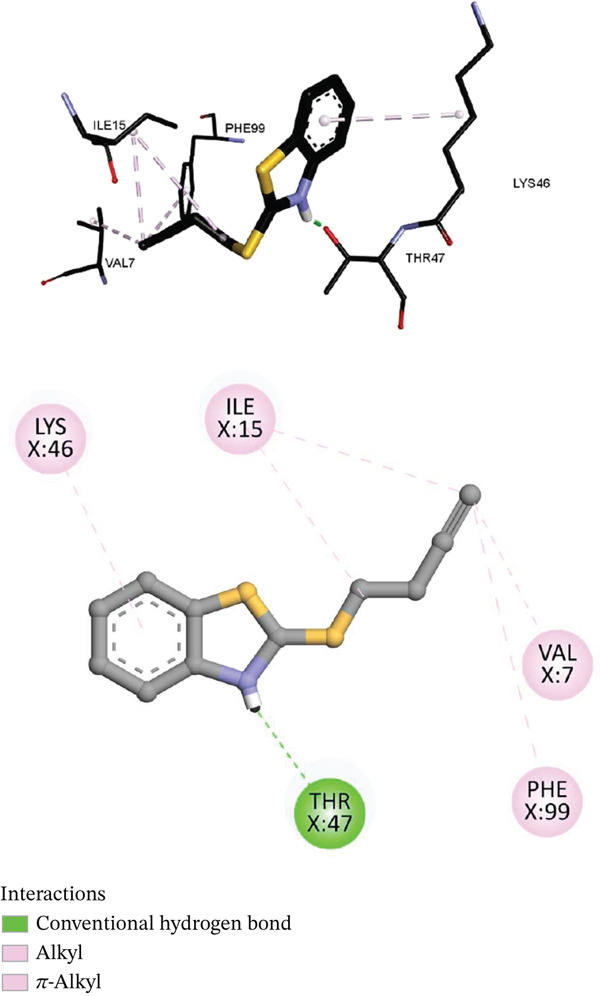
Docking pose of Compound 6 within *S. aureus* DHFR (PDB ID 3SQY).

Figure 2 depicts the docking pose of Compound **6** within *S. aureus* DHFR (PDB 3SQY), showing hydrogen bonding with PHE93 and *π*–*π* stacking with PHE99 anchoring the ligand in the NADPH‐binding region.

These complexes closely mimic the binding patterns of known inhibitors such as trimethoprim and methotrexate [57, 58], where the benzothiazolium ring contributes cation–*π* interactions with aromatic residues (PHE, TRP, and TYR). Tellurium enhances dispersion and polarizability, strengthening weak electrostatic contacts with O‐ and S‐containing residues. These effects are consistent with the moderate yet reproducible MIC values (31.25–125 *μ*g/mL) observed experimentally.

Docking data reveal a clear structure–activity relationship governed by the geometry and electronic distribution of the chalcogen‐linked substituent. Compound **4** (thiopentenyl), featuring an sp^2^‐hybridized C = C fragment, exhibits flexibility and moderate polarity—properties that favor interaction with larger, adaptive enzyme cavities such as DHFR. Conversely, Compound **6** (thiobutynyl), with its linear sp‐hybridized C ≡ C fragment, is more rigid and electronically delocalized, fitting snugly into the narrow ATP groove of GyrB with minimal conformational strain.

This distinction explains the selective affinity observed: The alkyne motif of Compound **6** preferentially targets GyrB, whereas the alkenyl fragment of Compound 4 aligns well with DHFR. The positively charged benzothiazolium ring further enhances binding through cation–*π* interactions [61], selectively recognizing aromatic clusters that are characteristic of bacterial—but not human—enzymes. The tellurium atom, as a heavy chalcogen, adds strong polarization and *σ*‐hole bonding potential with nucleophilic residues [59].

Overall, computational and experimental evidence converge on a mechanism involving the simultaneous inhibition of DNA topoisomerase II (GyrB) and folate metabolism (DHFR). This combined interference disrupts replication and redox balance, thereby explaining the pronounced antibiofilm activity of these benzothiazole derivatives, since suppression of both processes is essential for the eradication of sessile microbial populations [62].

## 4. Conclusion

The present study demonstrates that tellurium‐containing benzothiazole derivatives constitute a new class of multifunctional antibacterial agents capable of interfering with essential enzymatic processes in pathogenic bacteria. Experimental screening confirmed inhibitory activity against both *S. aureus* and *E. coli*, while molecular docking revealed that Compounds 4 (thiopentenyl) and 6 (thiobutynyl) interact selectively with two key bacterial enzymes—DNA GyrB and DHFR.

The alkynyl‐bearing Compound **6** aligns linearly within the narrow ATP‐binding groove of GyrB, forming stable hydrogen bonds and hydrophobic contacts reminiscent of coumarin‐like inhibitors. In contrast, the alkenyl Derivative **4** exhibits greater conformational adaptability, allowing favorable accommodation in the DHFR active site through hydrogen bonding and *π*–*π* stacking interactions with aromatic residues such as PHE and TRP. These complementary binding preferences explain the broad‐spectrum and antibiofilm efficacy observed experimentally.

The incorporation of tellurium atoms plays a decisive structural and electronic role, enhancing molecular polarizability and enabling stronger dispersive and charge–transfer interactions relative to sulfur analogues. In combination with the positively charged benzothiazolium core, these effects promote cation‐*π* stabilization within enzyme pockets, contributing to consistent affinity across both Gram‐positive and Gram‐negative bacteria.

Overall, the findings support an inhibitory mechanism—simultaneous blockade of DNA supercoiling via GyrB and disruption of folate metabolism through DHFR inhibition. This multitarget strategy underlies the strong antibiofilm performance and suggests that organotellurium heterocycles may serve as promising scaffolds for next‐generation antimicrobials capable of overcoming resistance. Future studies will involve molecular dynamics validation, cytotoxicity evaluation, and rational modification of substituent patterns to enhance selectivity and pharmacokinetic properties.

## Author Contributions

**Olha Haleha:** conceptualization; methodology; investigation; data curation; formal analysis. **Valeriy Pantyo:** methodology; investigation; validation; formal analysis; resources; writing—review & editing. **Nataliya Korol:** conceptualization; methodology; project administration; validation; visualization; writing—original draft. **Elvira Danko:** investigation; validation; resources; data curation; writing—review & editing. **Mykhailo Onysko:** conceptualization; methodology; funding acquisition; project administration; writing—review & editing.

## Funding

This study was supported by the National Research Foundation of Ukraine, № 2023.03/0176 «Strategy for directed synthesis of functional chalcogen halide materials for medical and energy needs».

## Disclosure

The authors take full responsibility for the integrity, originality, and scientific content of the manuscript.

## Conflicts of Interest

The authors declare no conflicts of interest.

## Supporting information


**Supporting Information** Additional supporting information can be found online in the Supporting Information section. NMR spectra (^1^H, ^13^C) of all synthesized compounds (PDF).

## Data Availability

The data used to support the findings of this study are included within the article.
