# Supplementary figures and images for: Synthesis, Biological Evaluation, and Molecular Docking of Tellurium‐Containing Benzothiazole Derivatives as Enzyme Inhibitors of DNA Gyrase and Dihydrofolate Reductase
Source: Biomed Res Int. 2026 Apr 16;2026:9439665. doi: 10.1155/bmri/9439665 (PMC13084523; doi:10.1155/bmri/9439665)

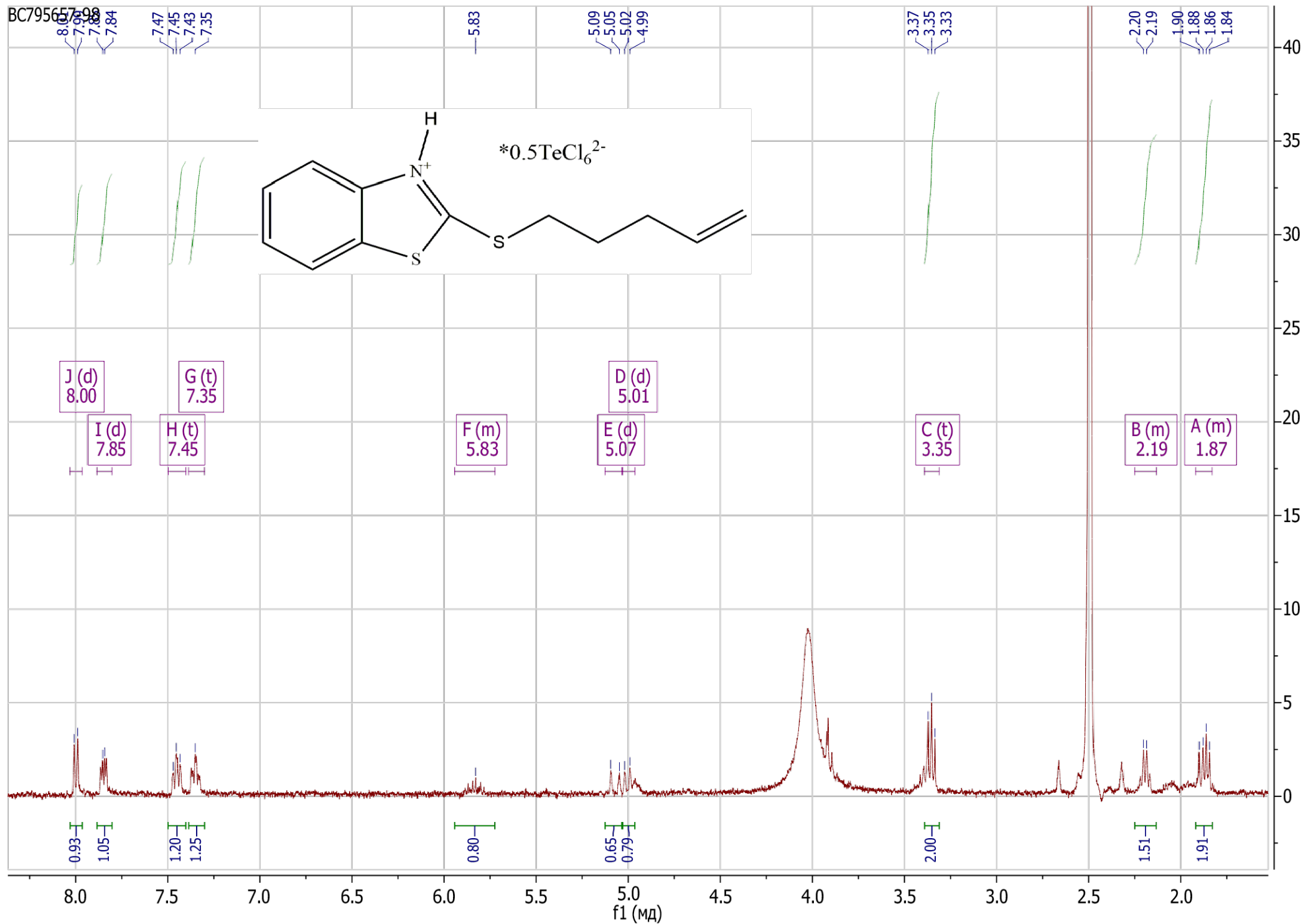

BG261936-15\_C13

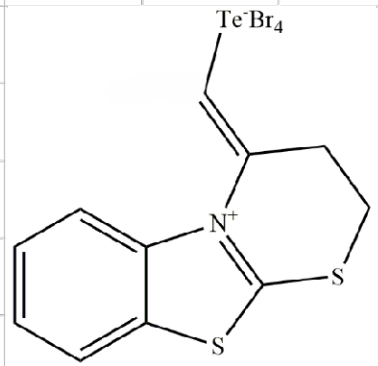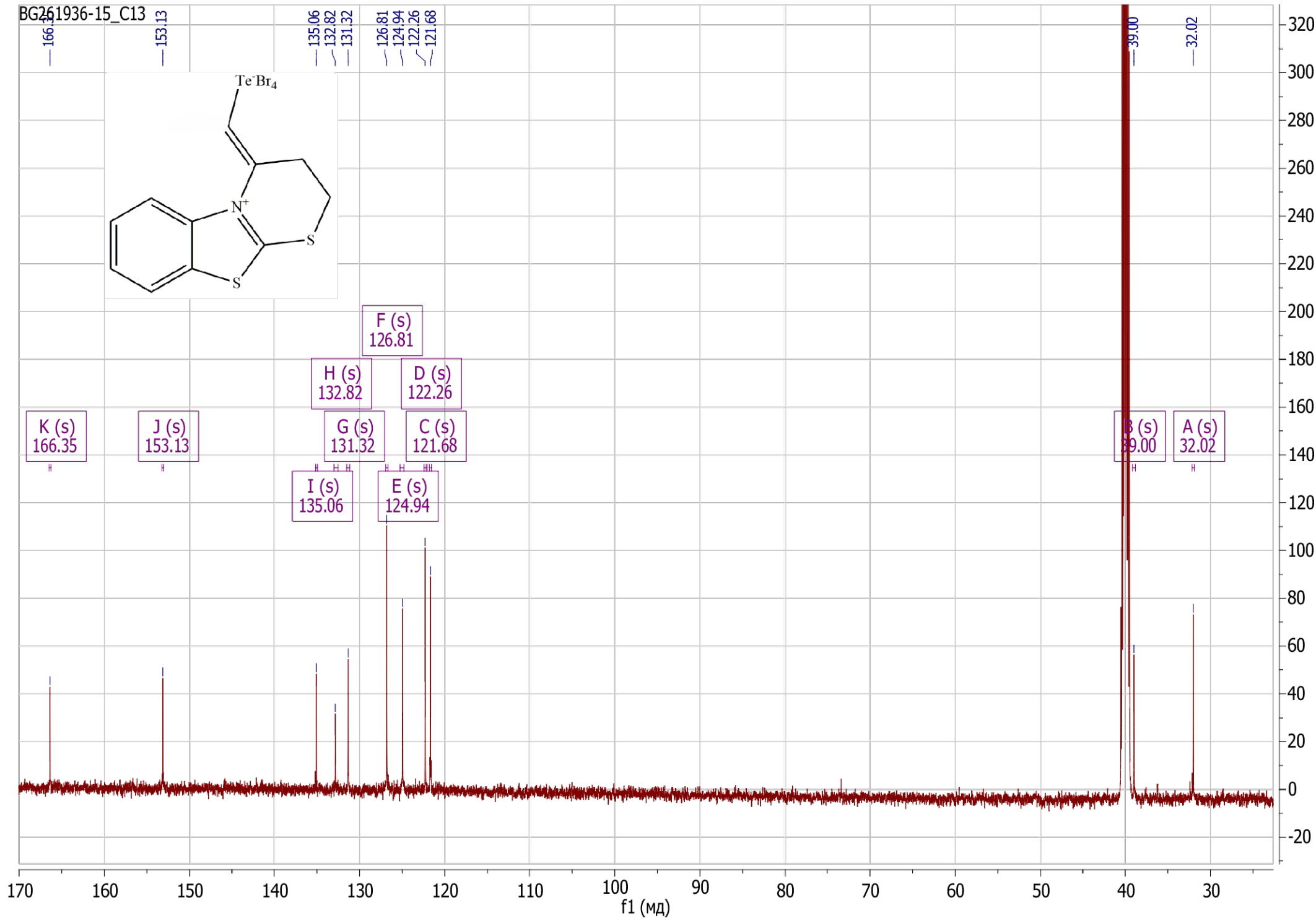

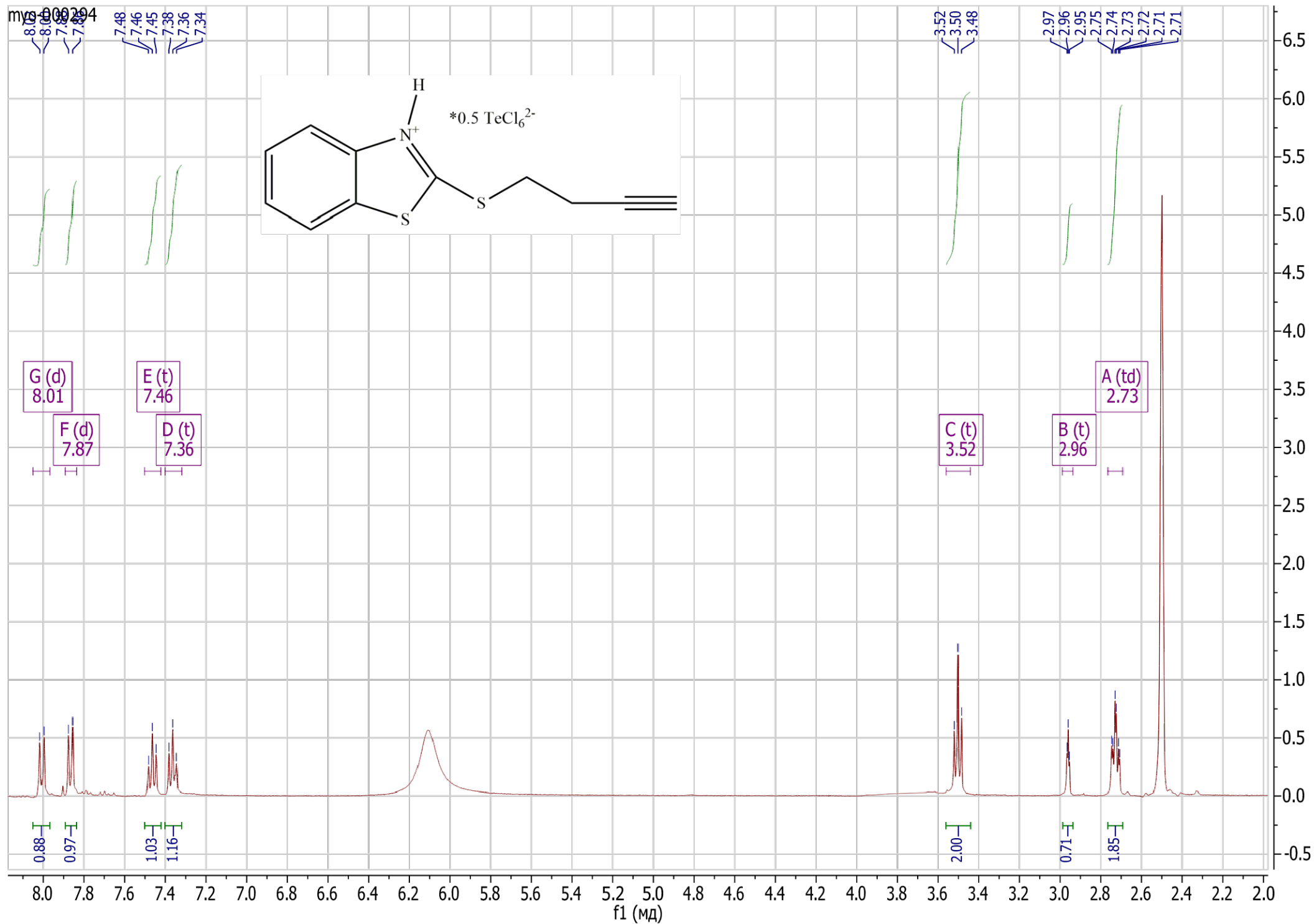

BD355076-29

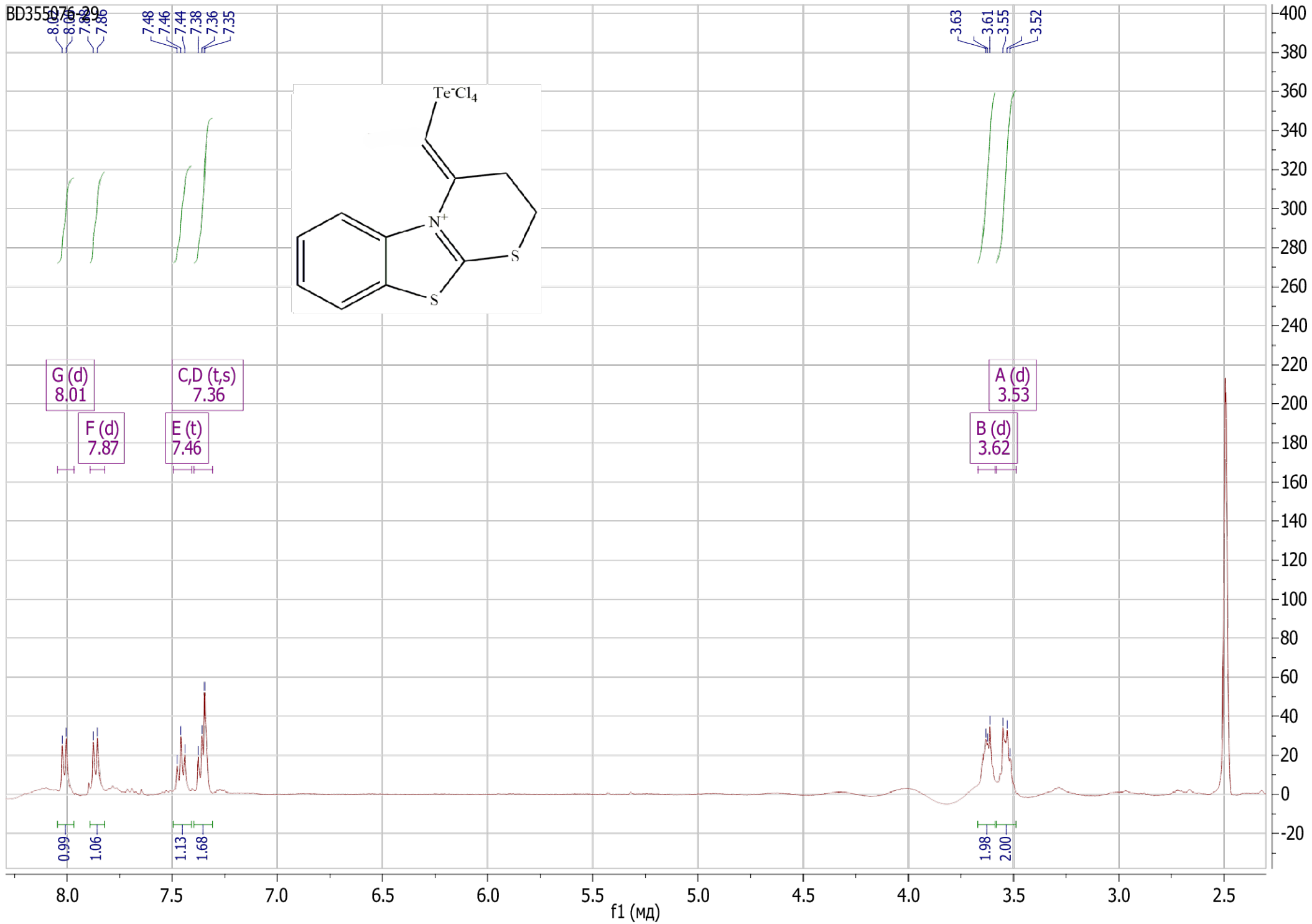

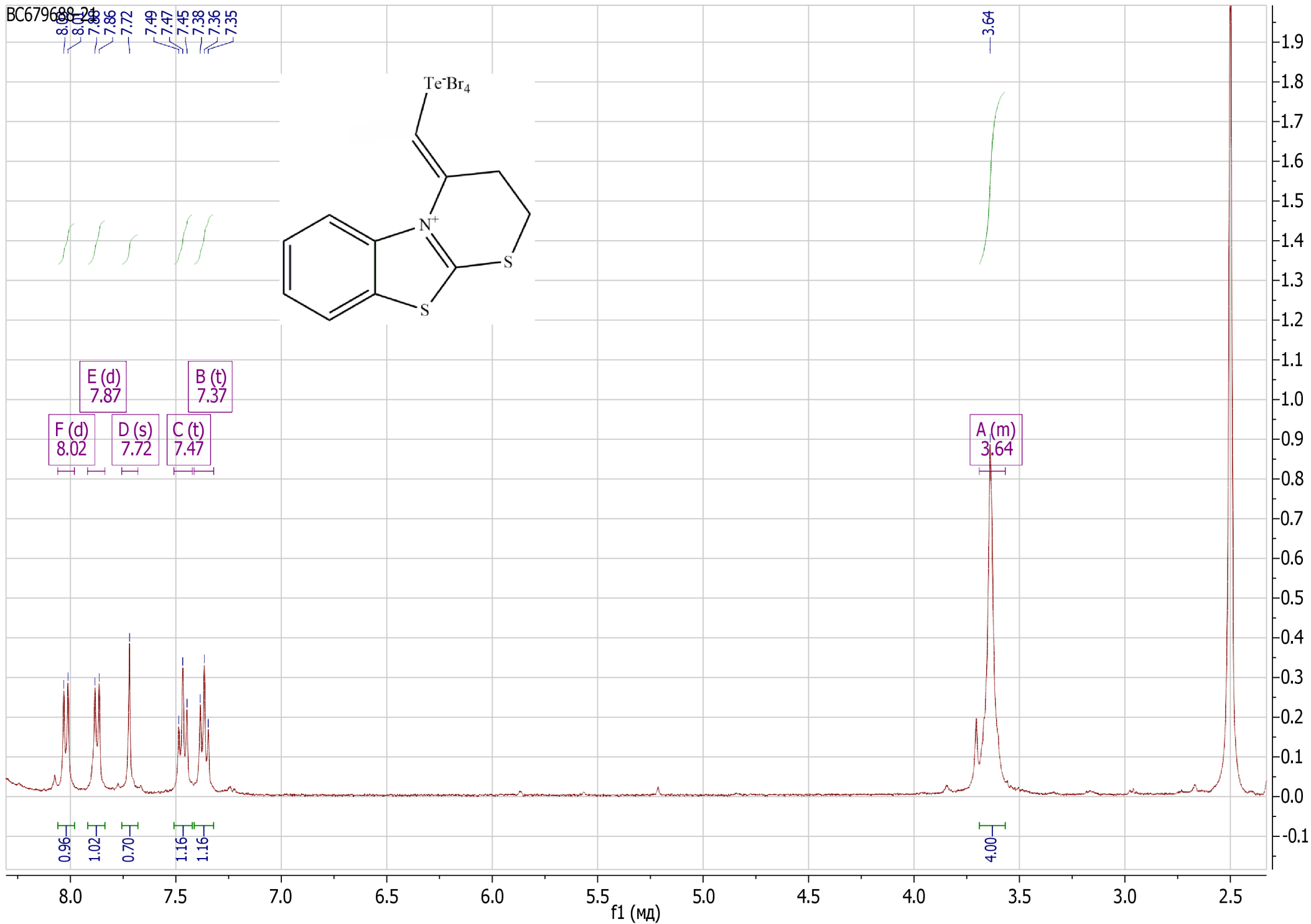

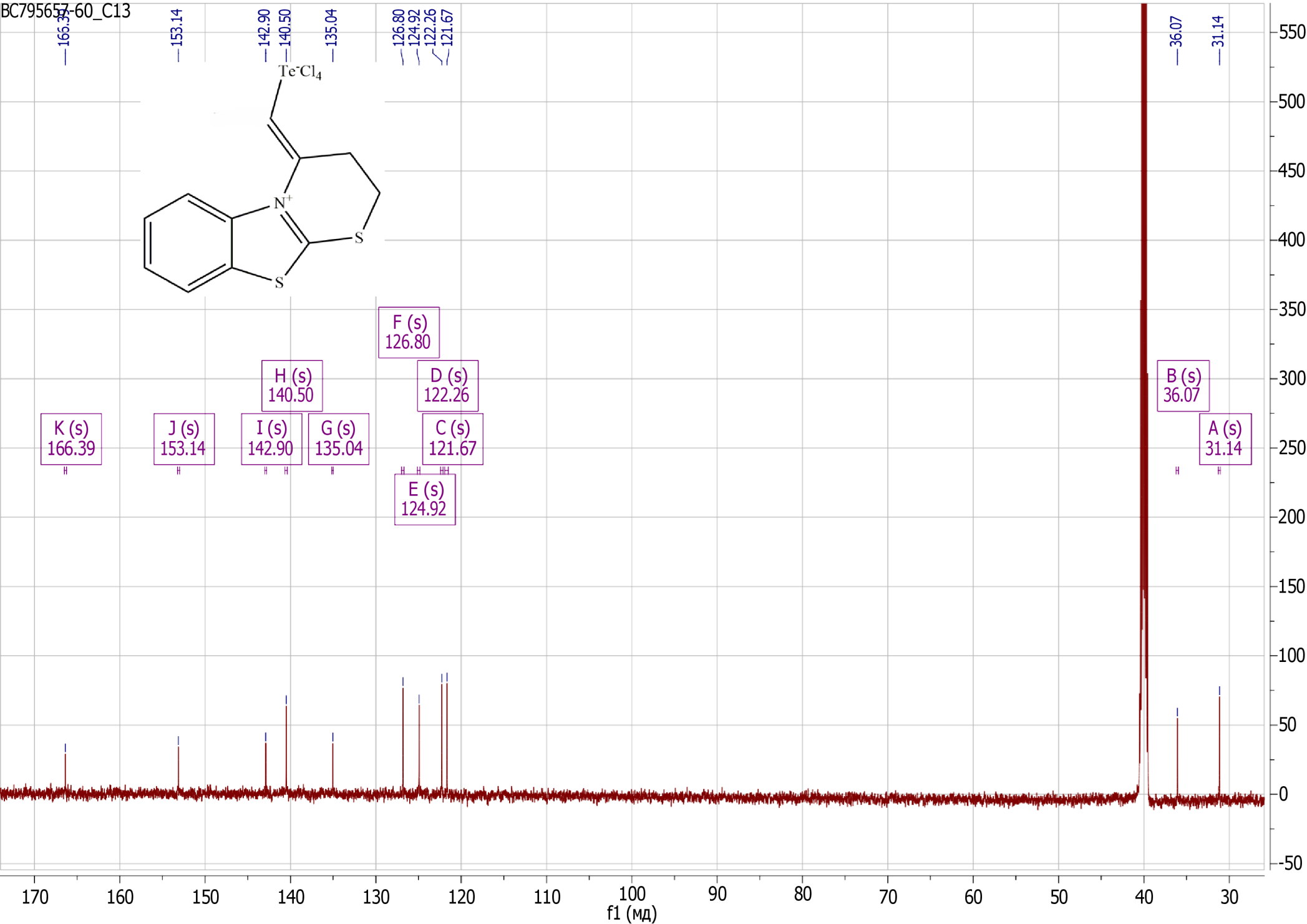

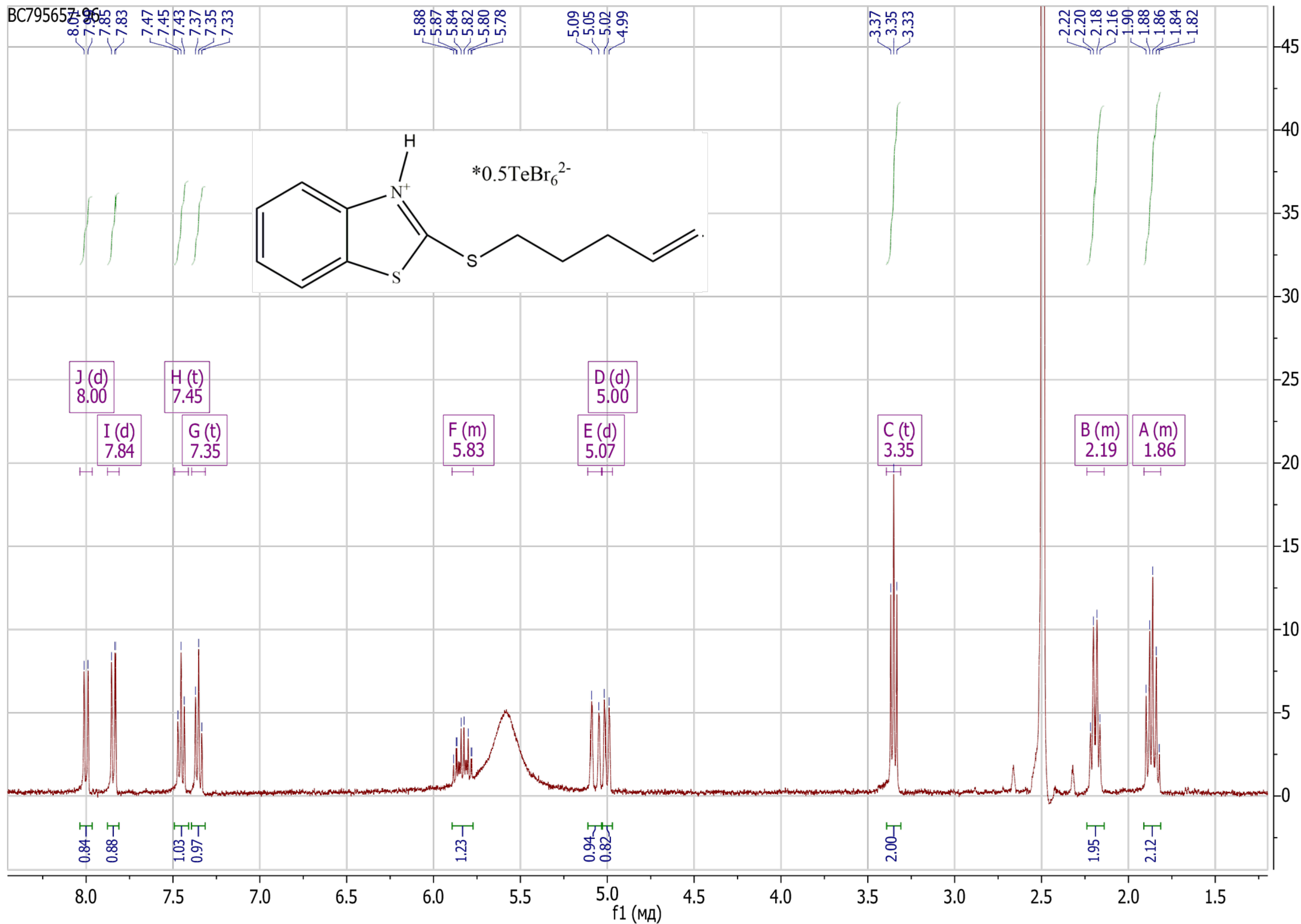

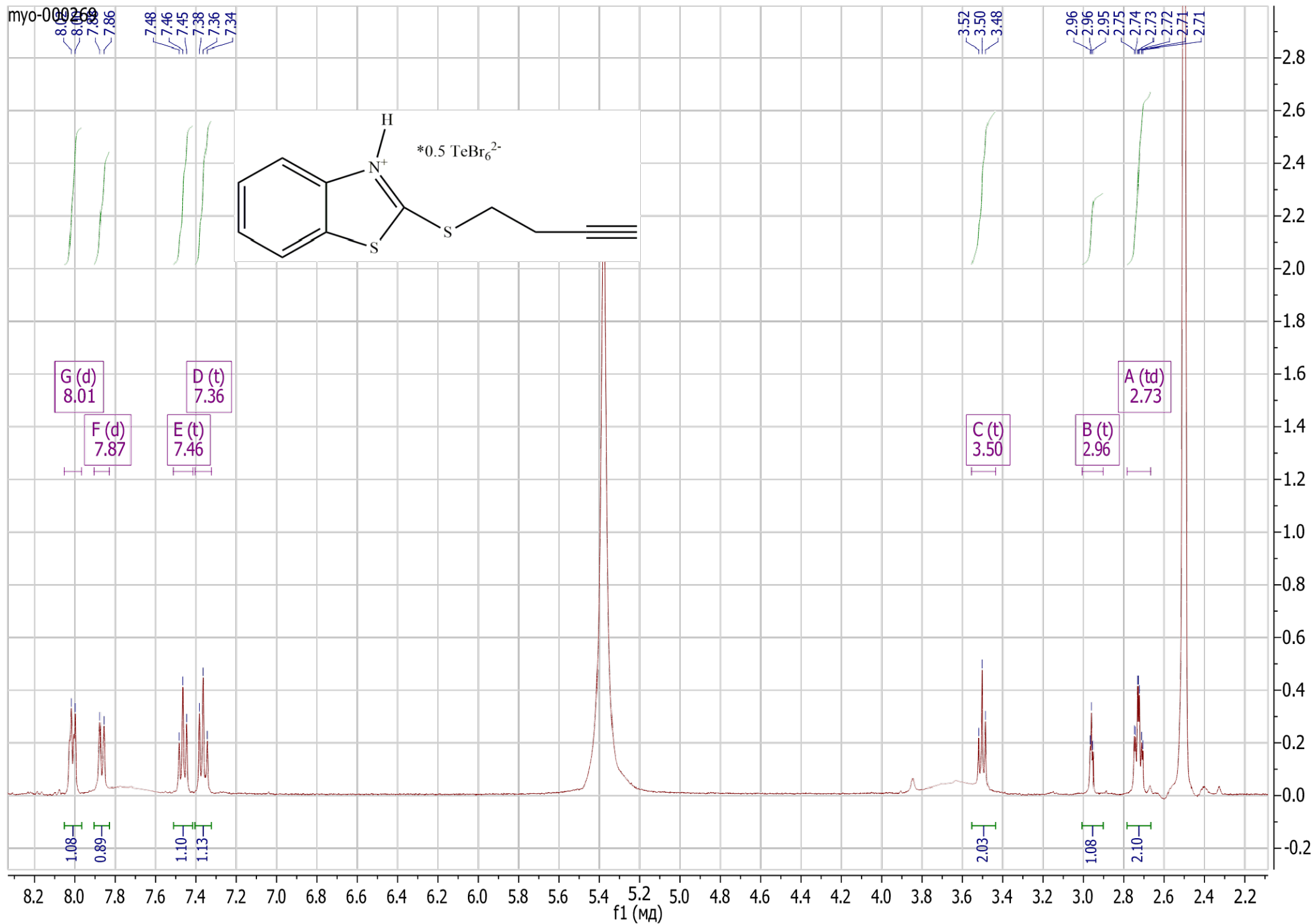

Supplement: Supplementary file 1 — Supporting Information Additional supporting information can be found online in the Supporting Information section. NMR spectra (1H, 13C) of all synthesized compounds (PDF). [file BMRI-2026-9439665-s001.pdf]
